# Clinical Utility of ^18^F-FDG PET in Neuroendocrine Tumors Prior to Peptide Receptor Radionuclide Therapy: A Systematic Review and Meta-Analysis

**DOI:** 10.3390/cancers13081813

**Published:** 2021-04-10

**Authors:** Emmanouil Alevroudis, Maria-Eleni Spei, Sofia N. Chatziioannou, Marina Tsoli, Göran Wallin, Gregory Kaltsas, Kosmas Daskalakis

**Affiliations:** 1 2nd Department of Radiology, Nuclear Medicine Unit, National and Kapodistrian University of Athens, General University Hospital Attikon, 11527 Athens, Greece; alevroudis@gmail.com (E.A.); sofiac@med.uoa.gr (S.N.C.); 2 1st Department of Propaedeutic Internal Medicine, Endocrine Unit, National and Kapodistrian, University of Athens, 11527 Athens, Greece; marilena_0108@hotmail.com (M.-E.S.); martso.mt@gmail.com (M.T.); gkaltsas@med.uoa.gr (G.K.); 3 Nuclear Medicine Division, Biomedical Research Foundation Academy of Athens, 4 Soranou Efesiou St., 11527 Athens, Greece; 4 Department of Surgery, Faculty of Medicine and Health, Örebro University, 701 85 Örebro, Sweden; goran.wallin@regionorebrolan.se

**Keywords:** neuroendocrine neoplasm, ^18^F-FDG PET, PRRT

## Abstract

**Simple Summary:**

Functional imaging with ^18^F-fluorodeoxyglucose positron emission tomography (^18^F-FDG PET) has evolved into a major clinical tool in cancer diagnosis and management for many malignancies in diverse clinical settings, providing valuable information on tumor behavior and aggressiveness. In the field of neuroendocrine tumors (NETs), recent advances in molecular imaging and targeted treatments with novel theranostic agents favor a more patient-tailored approach. Although peptide receptor radionuclide therapy (PRRT) has recently become an established therapy for progressive NETs, the role of ^18^F-FDG PET prior to PRRT in patients with NETs of different origins and grades remains to be determined. Herein, we provide a comprehensive summary of available evidence in contemporary literature by means of a systematic review and meta-analysis, demonstrating that dual-functional imaging with ^68^Ga-DOTA-peptides and ^18^F-FDG prior to PRRT appears to be a useful tool in NET management by delineating tumor somatostatin receptor expression and glycolytic metabolic activity, and predicting tumor response and survival outcomes.

**Abstract:**

The role of ^18^F-FDG PET in patients with variable grades of neuroendocrine tumors (NETs) prior to peptide receptor radionuclide therapy (PRRT) has not been adequately elucidated. We aimed to evaluate the impact of ^18^F-FDG PET status on disease control rate (DCR), progression-free survival (PFS), and overall survival (OS) in neuroendocrine tumor (NET) patients receiving PRRT. We searched the MEDLINE, Embase, Cochrane Library, and Web of Science databases up to July 2020 and used the Newcastle-Ottawa scale (NOS) criteria to assess quality/risk of bias. A total of 5091 articles were screened. In 12 studies, 1492 unique patients with NETs of different origins were included. The DCR for patients with negative ^18^F-FDG PET status prior to PRRT initiation was 91.9%, compared to 74.2% in patients with positive ^18^F-FDG PET status (random effects odds ratio (OR): 4.85; 95% CI: 2.27–10.36). Adjusted analysis of pooled hazard ratios (HRs) confirmed longer PFS and OS in NET patients receiving PRRT with negative ^18^F-FDG PET (random effects HR:2.45; 95%CIs: 1.48–4.04 and HR:2.25; 95% CIs:1.55–3.28, respectively). In conclusion, ^18^F-FDG PET imaging prior to PRRT administration appears to be a useful tool in NET patients to predict tumor response and survival outcomes and a negative FDG uptake of the tumor is associated with prolonged PFS and OS.

## 1. Introduction

Neuroendocrine neoplasms (NENs) are a group of biologically and clinically diverse neoplasms arising from the diffuse neuroendocrine system. They are mainly located in the gastroenteropancreatic and bronchopulmonary systems, and less commonly in other sites. NENs are very heterogeneous tumors that can be categorized clinically by primary origin, differentiation, Ki67 proliferation index, somatostatin receptor (SSTR) expression, the extent of metastatic spread, and secretory status. Biologically, NENs are also increasingly categorized by mutational patterns and gene-expression profiles [1]. Significant differences in survival are generally found among various primary tumor sites, while many NEN patients are diagnosed at a late stage, when locoregional and/or distant metastases have already occurred [2].

The recently updated grading nomenclature by the World Health Organization (WHO) proposes a framework for the universal classification of neuroendocrine neoplasia [3]. NENs may be well (neuroendocrine tumors; NETs) or poorly differentiated (neuroendocrine carcinomas; NECs), with diverse incidence and prevalence in different organs. In addition, the WHO taxonomy into NETs and NECs is supported by genetic evidence and clinical, epidemiologic, histologic, and prognostic differences [4]. Across different organ systems, NETs are graded as G1, G2, or G3 based on mitotic count and/or Ki-67 labeling index, and/or the presence of necrosis, whereas NECs are commonly considered as high-grade neoplasms [5]. Importantly, the Ki67 index has been established as one of the most reliable factors in the prognostic evaluation of gastroenteropancreatic NENs [6]. With respect to the newly introduced category of G3 NET, these tumors are less aggressive than NECs, but show a worse survival outcome than NET G2 [7,8,9].

Imaging and treatment with novel theranostic agents offer a patient-tailored approach with recent advances aiming to enhance the effectiveness of targeted treatments.Peptide receptor radionuclide therapy (PRRT) is an established therapy for NETs that takes advantage of the overexpression of somatostatin receptors (SSTRs) on the NET cell surface, to vehicle radioactivity to neoplastic tissues [10]. After the favorable outcomes presented in the NETTER-1 trial, PRRT has indeed gained popularity as the method of choice for NET treatment and is currently an established therapeutic option for progressive gastroenteropancreatic NETs after 1st or 2nd line treatment [11]. Although, PRRT is also registered for broncopulmonary and thymic NETs in the US, following promising results in studies on these patient subsets, it is not currently approved for non gastroetero-pancreatic NETs in many countries [12,13,14,15,16,17]. In addition, PRRT has been assessed and could be an effective therapy in a more heterogeneous group of SSTR-expressing tumors, consisting of pheochromocytomas and paragangliomas, medullary thyroid carcinomas, and meningiomas [18].

Notably, a necessary condition for a patient’s PRRT eligibility is finding the existence of increased overexpression of SSTRs in functional imaging. Importantly, with respect to tumor grading, the biopsy material is usually received from a unique point of a lesion and may not always be representative of the tumor aggressiveness. Nevertheless, many tumor clones with variable biological behaviors often coexist at different foci. Hence, it may not be adequate to provide a complete functional frame of the disease based on tumor grading and SSTR imaging [19]. Therefore, in recent years, a combined approach of nuclear medicine imaging with ^68^Ga-DOTA-TOC/TATE/NOC-PET and ^18^F-FDG-PET scan has been proposed [20]. In particular, the ^68^Ga-DOTA-TOC/TATE/NOC-PET scan can detect lesions that overexpress SSTRs while the ^18^F-FDG-PET scan reveals alterations with increased glycolytic metabolism. The combination of these imaging modalities prior to PRRT administration would enable a complete mapping of the disease with a relatively small radial load for the patient [21,22]. Nevertheless, it is frequently encountered, especially in G2 NETs, positive findings in both the aforementioned PET scans, with the administration of PRRT in this scenario still being advisable under the condition of anatomical agreement of the gallium and glucose uptake lesions [23].

Although it is generally accepted that upregulated glycolytic activity is an unfavorable prognostic factor associated with a more aggressive clinical course, studies accessing the PRRT response in relation to the ^18^F-FDG-PET status at baseline are rather limited. The aim of this systematic review and meta-analysis was to evaluate differences in the efficacy of PRRT between baseline ^18^F-FDG positive NET patients vs. negative ones and determine whether ^18^F-FDG PET could be used prior to PRRT administration to predict treatment responses as well as progression-free (PFS) and overall survival (OS) outcomes.

## 2. Material and Methods

### 2.1. Study Selection

Retrospective and prospective single- and multi-center cohort studies on patients with NENs receiving PRRT were assessed for eligibility. The following outcomes were required for eligibility: disease control rate according to RECIST or SWOG criteria, PFS and OS following PPTR. Surgical series with a sample size of at least 10 NET patients undergoing PPTR was necessary for study inclusion. Among multiple reports from the same institution with an overlap in patient cohorts of two studies, the latest eligible study was selected, unless these studies referred to different patient groups or time periods. We followed the PRISMA guidelines for reporting the results of the study [24].

### 2.2. Search Strategy

We conducted a systematic search in the Medline, Embase, Cochrane Library, and Web of Science databases for published and unpublished reports (conference abstracts) up to July 2020 in any language to determine eligible studies. The electronic search strategy we applied is described in the supplemental data section (Table S1). We examined full manuscripts of potentially eligible studies as necessary to finalize the study selection. Two of the authors (E.A. and K.D.) independently evaluated the eligible articles for relevance to the planned scope of the review. Reference lists of key publications were also reviewed for eligibility.

### 2.3. Data Extraction

Data used in this systematic review and meta-analysis were independently extracted by the authors E.A. and K.D. The primary outcome was defined as DCR and PFS associated with ^18^F-FDG avidity prior to PRRT in NETs and the secondary outcome was the overall mortality of NET patients in this setting. We formulated the study hypothesis before data collection and resolved any discrepancies concerning the extracted data by consensus between E.A. and K.D.

### 2.4. Risk of Bias

We classified the included single- and multi-center institutional studies using the classical epidemiologic study design of cohort studies [25]. The Newcastle-Ottawa scale (NOS) template was used for quality/risk of bias assessment of the included studies [26]. The score derived from NOS application ranged from 0 to 9 (worst to best). We assigned lower NOS scores to studies with a small sample size, ambiguity over NET inclusion criteria, inadequate follow-up, and lack of clarity over standardized uptake value (SUV) cut-offs in respect to ^18^F-FDG positivity.

### 2.5. Statistical Analysis and Exploration of Heterogeneity

The pooled estimate for the association of ^18^F-FDG avidity in NET patients prior to PRRT with the outcome of interest was evaluated by combining the study-specific odds ratios (ORs) and hazard ratios (HRs) with random effects in the presence of heterogeneity. With respect to DCR, we calculated the ORs taking into account the correction of Haldane-Anscombe about 0 cells [27]. The random variance component was estimated using the approach by Der Simonian and Laird [28]. To explore heterogeneity between the studies the I^2^ statistics were used. When I^2^ was > 0.50% the statistical heterogeneity was considered substantial [29]. Publication bias was assessed by Egger’s test and funnel plots that were used to investigate the asymmetry among the study estimates [30,31]. All the analyses were performed using the STATA statistical package (version 13.1; StataCorp, College Station, TX, USA).

## 3. Results

### 3.1. Characteristics of Included Studies

**Figure 1:**
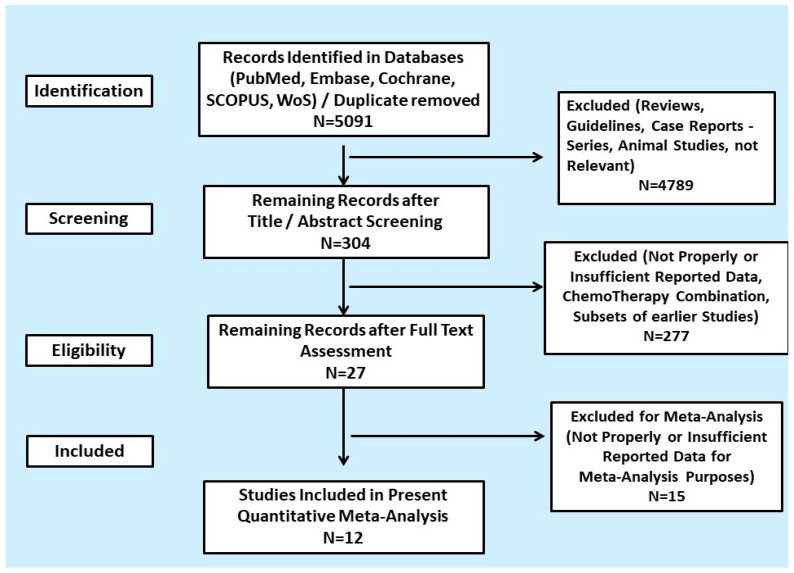
Preferred reporting items for systematic reviews and meta-analyses (PRISMA) flow diagram of the systematic review and quantitative meta-analysis.

A total of 5091 articles and conference abstracts were screened. From 12 studies, 1492 unique patients with NET who underwent ^18^F-FDG PET imaging prior to PRRT were included. The PRISMA flow diagram of the study is presented in Figure 1. The systematic literature search strategy is presented in Table S1 and the characteristics of the included studies in Table 1.

Five studies in our meta-analysis included patients of G1 and G2 only [32,33,34,35]; (and seven studies included NET patients of all grades [12,36,37,38,39,40,41]. Overall, 491 patients with G1 (32.9%), 720 patients with G2 (48.2%), 63 patients with no clear distinction between G1 and G2 (4.2%), 85 patients with G3 (5.7%), and 133 patients with an unspecified grade (8.9%; among these were also patients with mediastinal NETs) were included in the present systematic review and meta-analysis (Table 1).

### 3.2. Quality and Risk of Bias Assessment

The Newcastle–Ottawa scale (NOS) star template for quality assessment of each study is presented in Table S2. No randomized controlled trials were identified. We included two prospective and ten retrospective observational cohort studies based on single- or multi-center institutional data. The included studies achieved moderate to high quality of evidence.

### 3.3. Pooled Results for Disease Control Rates following Peptide Receptor Radionuclide Therapy

**Figure 2:**
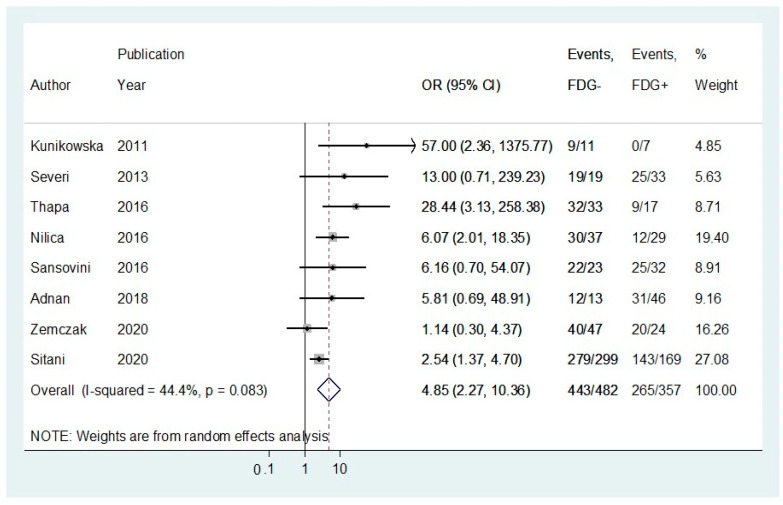
Forest plot comparing odds ratios of disease control rate (DCR) in ^18^F-FDG (−) vs. ^18^F-FDG (+) NET patients receiving peptide receptor radionuclide therapy (PRRT). Meta-analysis carried out using a random-effects model; odds ratios are shown with 95% confidence intervals.

**Figure 3:**
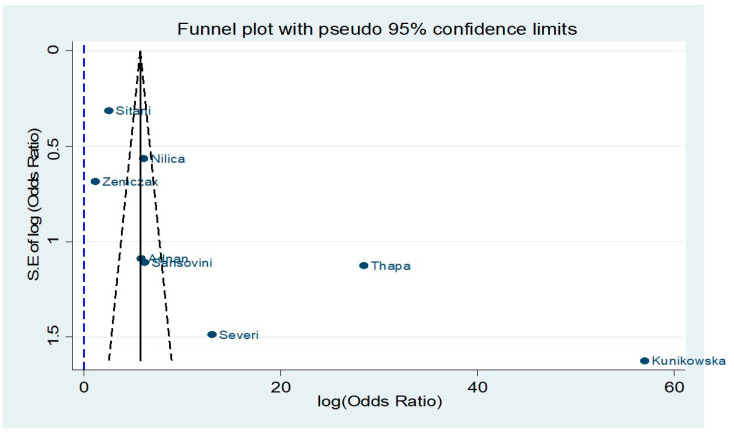
Funnel plot for studies included in the comparison of ^18^F-FDG(−) vs. ^18^F-FDG(+) NET patients receiving peptide receptor radionuclide therapy (PPTR) with respect to disease control rate (DCR).

Eight studies reported DCR following PRRT and were stratified by ^18^F-FDG status using OR analysis [32,33,34,35,36,37,39,42]. The DCR rate for patients with a negative ^18^F-FDG status prior to PRRT initiation was 91.9%, compared to 74.2% in patients with positive ^18^F-FDG imaging (Figure 2; random effects OR:4.85; 95%CI: 2.27–10.36; Figure 2). There was no significant heterogeneity across the studies (I^2^ = 44.4%, *p*-value > 0.05). A funnel plot (Figure 3) was also produced with some evidence of asymmetry. However, Egger’s test (*p*-value > 0.05) and Galbraith’s plot (Figure S1A,B) showed no indication of publication bias or significant heterogeneity across the included studies.

### 3.4. Pooled Results for Adjusted Progression-Free Survival (PFS) Rates following Peptide Receptor Radionuclide Therapy

**Figure 4:**
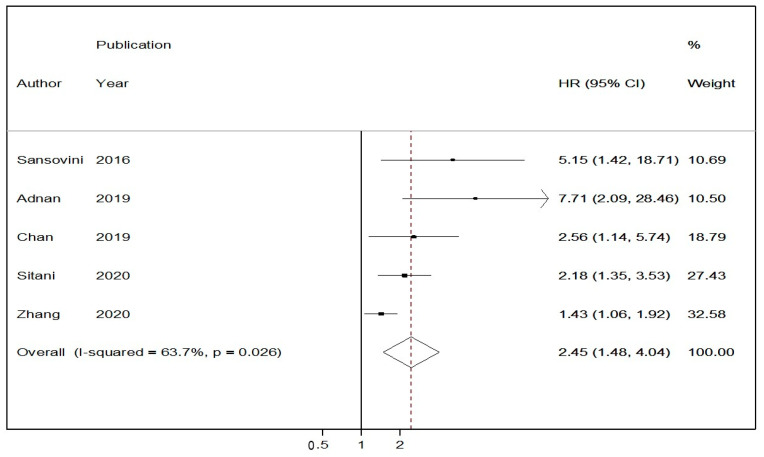
Forest plot comparing adjusted hazard ratios of progression-free survival in ^18^F-FDG (−) vs. ^18^F-FDG (+) NET patients receiving peptide receptor radionuclide therapy (PRRT). Meta-analysis carried out using a random-effects model; hazard ratios are shown with 95% confidence intervals.

**Figure 5:**
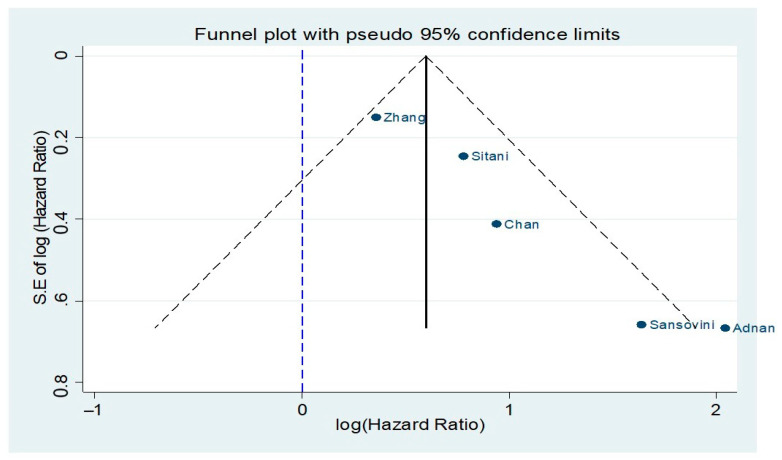
Funnel plot for studies included in the comparison of ^18^F-FDG (−) vs. ^18^F-FDG (+) NET patients receiving peptide receptor radionuclide therapy (PPTR) with respect to progression-free survival (PFS).

In five studies reporting Cox-regression multivariable PFS analyses following PRRT stratified by ^18^F-FDG avidity prior to treatment initiation, a random-effects HR of 2.45 (95% CIs:1.48–4.04) was demonstrated in the ^18^F-FDG positive group vs. the ^18^F-FDG negative one (Figure 4) [12,33,38,39,40]. There was significant heterogeneity across the studies (I^2^ = 63.7%, *p*-value < 0.05). A funnel plot (Figure 5) was also produced with some evidence of asymmetry. Egger’s test (*p*-value < 0.05) and Galbraith’s plot (Figure S2A,B, respectively) pointed towards publication bias and/or significant heterogeneity across the included studies; however, the number of included studies in this adjusted HR meta-analysis model was limited.

### 3.5. Pooled Results for Adjusted Overall Survival (OS) Rates following Peptide Receptor Radionuclide Therapy

**Figure 6:**
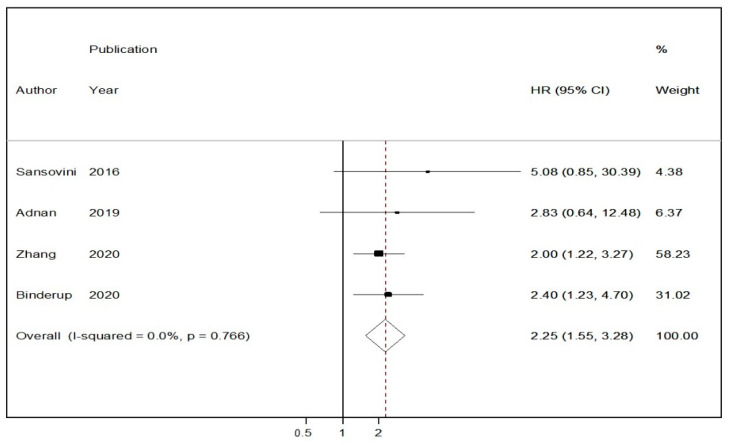
Forest plot comparing adjusted hazard ratios of overall mortality in ^18^F-FDG (−) vs. ^18^F-FDG (+) NEN patients receiving peptide receptor radionuclide therapy (PRRT). Meta-analysis carried out using a random-effects model; hazard ratios are shown with 95% confidence intervals.

In four studies reporting Cox-regression multivariable survival analyses following PRRT stratified by ^18^F-FDG avidity, a random-effects model HR of 2.25 (95% CIs: 1.55–3.28) was demonstrated in the ^18^F-FDG positive vs. the ^18^F-FDG negative group (Figure 6) [12,33,38,41]. There was no evidence of heterogeneity across the studies (I^2^ = 0%, *p*-value > 0.10)

**Figure 7:**
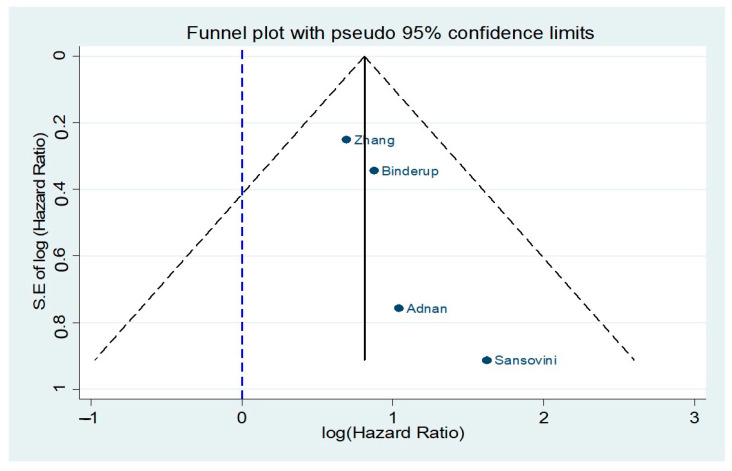
Funnel plot for studies included in the comparison of ^18^F-FDG (−) vs. ^18^F-FDG (+) NET patients receiving peptide receptor radionuclide therapy (PPTR) with respect to overall survival (OS).

A funnel plot (Figure 7) was also produced with no apparent evidence of asymmetry. Egger’s test (*p*-value > 0.05, Figure S3) showed no indication of publication bias among the included studies.

## 4. Discussion

Our systematic review and quantitative meta-analysis demonstrate that DCR in patients with NET receiving PRRT with a negative ^18^F-FDG PET prior to treatment initiation was 91.9%, vs. 74.2%, in patients with a positive ^18^F-FGD PET (OR: 4.85; 95%CI: 2.27–10.36). Adjusted HR multivariable PFS analysis revealed that patients with positive ^18^F-FDG PET prior to PRRT initiation had a 2.9-fold increased risk of progression compared to NEN patients with a negative ^18^F-FDG PET. In addition, adjusted HR multivariable OS analysis confirmed an approximately 2.3-fold increased risk of overall mortality in NET patients with a positive ^18^F-FDG PET prior to PRRT compared to patients with a negative one in this setting. Although, favorable outcomes in terms of treatment response and disease stabilization following PRRT were evident in both ^18^F-FDG negative and positive NETs with overexpression of SSTRs, patients with ^18^F-FDG PET positive tumors had a higher risk for progression and death following PRRT administration. Therefore, ^18^F-FDG PET may be utilized as a predictive tool in NET patients receiving PRRT.

All the included studies achieved moderate to high quality by scoring ≥ seven stars in the NOS star template. With regards to pooled DCR meta-analysis, the presence of certain asymmetry in the funnel plot and its narrow shape could potentially indicate a small study bias and differences in the design of the included studies. Although we encountered moderate inter-study heterogeneity in this sub-analysis, as high as 44%, it did not reach statistical significance. In addition, the studies at the bottom of the graph mainly contributing to the observed asymmetry had quite different numbers of ^18^F-FDG-positive cases vs. negative ones and a higher risk of bias with lower scores in the comparability assessment in the NOS template (Table S2) [34,37,42]. Finally, the number of studies included in our meta-analyses is relatively small, so there is a high probability that departures from the ideal funnel shape may occur due to chance.

Inter-study heterogeneity was observed in the unadjusted HR meta-analysis for PFS. Complementary testing revealed potential publication bias and between-study heterogeneity in our meta-analysis with respect to PFS outcomes. The study by Adnan et al. on primaries with more aggressive biological behavior, i.e., metastatic NETs of the mediastinum, contributed the most to the interstudy heterogeneity in this analysis [12]. Importantly, the studies included in our meta-analysis lacked the granularity to identify certain subsets of patients who may derive the most benefit from the utilization of ^18^F-FDG PET imaging prior to PRRT administration. For example, data on Ki-67 proliferation index in relation to ^18^F-FDG status, discrepancies in liver tumor burden, and the presence of extrahepatic metastases, as well as detailed data on prior surgical and systemic therapies were not available in all the included studies. In addition, as the clinical scenario for the medical management of metastatic NENs may have changed during part of the time for the studies included in our meta-analysis, the strata of ^18^F-FDG PET groups that we compare may be heterogeneous and subjected to selection bias.

Generally, the primary tumor site does not seem to play a pivotal role in predicting PRRT response, although some studies provide evidence of more favorable outcomes following PRRT in patients with gastroenteropancreatic primaries, especially in those with small intestinal NETs [43]. With regards to NET origin in relation to ^18^F-FDG PET status and prediction to PRRT response, our meta-analysis included studies with a diverse spectrum of NET primaries, mainly gastroenteropancreatic and bronchopulmonary tumors as well as mediastinal ones, exhibiting a wide range of generally favorable PRRT responses, and variable PFS and OS outcomes. To reduce tumor heterogeneity, we did not include studies on medullary thyroid carcinoma, as well as studies on NECs. Four studies in our meta-analysis investigated a possible correlation between NET origin and PRRT efficacy [12,36,38,39]. However, due to differences in their design and the limited numbers of different NET primaries introduced in their survival analyses, no safe conclusion could be derived. Importantly, although the inclusion of different NET primaries may have introduced certain confounding elements to our results, higher HRs for progression and death was evident across all NET primaries for ^18^F-FDG PET-positive tumors, also when comparing studies with gastroenteropancreatic NETs with those on bronchopulmonary and/or mediastinal primaries; thus, signifying that ^18^F-FDG PET is an important tool to delineate tumor aggressiveness and predict PRRT response, possibly resolving limitations associated with tumor heterogeneity.

Tumor grading is one of the most important independent predictors of OS in NEN patients [2,6]. Baum et al. retrospectively investigated PRRT efficacy in 1048 NET patients and demonstrated superior OS and PFS outcomes in G1 NETs as compared to G3 ones [44]. PRRT is currently approved for progressive G1 and G2 gastroenteropancreatic NETs with high uptake on ^68^Ga-DOTATATE, exhibiting favorable PRRT responses and prime survival outcomes, both in terms of PFS and OS [18]. Although patients with NET G3 have a more dismal prognosis and PRRT is not approved as of yet for these patients in most countries, promising results have been reported also for NET G3 in a large multicenter study by Carlsen and et al. [45], as well as in smaller studies [38,46,47,48,49,50]. Of note, the country-specific PRRT approval for higher grade NETs probably also affects the implementation of ^18^F-FDG PET in this setting. Therefore, the ongoing NETTER-2 trial, currently recruiting patients, to assess the efficacy of PRRT in NET G2 and G3 is eagerly anticipated. With regards to the Ki67 proliferation index in relation to ^18^F-FDG PET status in our meta-analysis, we provide some evidence that G1 or G2 patients may also have ^18^F-FDG-positive tumors initially or may develop ^18^F-FDG-positive lesions during follow-up with important implications in therapy optimization and disease surveillance. Although the majority of the included NEN patients were G1 and G2 (86.8%), the span of Ki67 proliferation index in G2 tumors is rather wide (3–20) and cases with a higher level of Ki67 within G2 may indeed exhibit substantial differences in baseline ^18^F-FDG PET status and diverse patient outcomes with respect to PRRT responses. In four studies included in our meta-analysis, lower grade NETs exhibited more favorable PRRT responses in terms of PFS and OS [34,35,38,41]. However, these results should also be interpreted in the light of a positive correlation between higher tumor grade and FDG positivity. Chan et al. reported that patients with a higher grade were more likely to demonstrate higher quantitative parameters in ^18^F-FDG PET imaging, such as metabolic tumor volume and total lesion glycolysis; however, PFS was not significantly affected by grade [40]. Importantly, although ^18^F-FDG PET positivity prior to PRRT administration was confirmed as an independent factor for PFS and OS in all the included studies undertaking multivariable Cox-regression analysis, tumor grade was only introduced in the regression model of two of the studies [38,41]. Therefore, no safe conclusions could be derived from our meta-analysis concerning the association between Ki67 proliferation index and baseline ^18^F-FDG PET status in patients being administered PRRT, as well as the impact of grading in patient outcomes in this setting.

Concerning the maximum standardized uptake value (SUVmax) of pathological lesions on ^18^F-FDG PET, the application of SUVmax cut-offs in the included studies to assess the binary outcomes of interest ranging from 2.5 to 5 (Table 1). A positive ^18^F-FDG PET with an SUV of 4.5 or greater has been confirmed as a poor prognostic factor for OS in patients with metastatic NETs [51,52]. However, as none of the studies reported patient data at the individual level, we were not able to perform a diagnostic test accuracy meta-analysis, i.e., apply a receiver-operating-characteristic (ROC) analysis to determine the positive threshold for SUV with respect to a prognostic and predictive evaluation. In two studies, SUVmax stratified analysis was presented applying the following strata: SUVmax < 5, SUVmax: 5–10, SUVmax > 10 with evidence of a negative correlation between higher lesional FDG uptake and PRRT responses/survival outcomes [12,32]. In addition, Binderup et al. demonstrated a higher risk of progression and death for patients receiving PRRT with a baseline SUVmax > 3 and > 9, respectively [53].

Although, stage IV NETs with increased SSTR expression may be good candidates for PRRT at some point in the course of the disease, chemotherapy and/or molecular targeted therapies seem to be the treatment of choice for the subset of NETs with the minimum expression of SSTR and higher glycolytic metabolism. However, due to NEN heterogeneity, both SSTR overexpression and increased glycolytic metabolism findings may be observed by ^68^Ga-DOTA-TOC/TATE/NOC-PET/CT scan and ^18^F-FDG-PET/CT, respectively. In these cases, PRRT administration is feasible, provided that foci anatomical concordance is noted in the two PET imaging modalities. Indeed, spatially discordant FDG-avid disease showing low or absent SSTR expression is commonly considered as an exclusion criterion for PRRT eligibility in most studies [22,47,50,54,55,56].

Furthermore, for patients whose dual-functional imaging scans may show increased SSTR density and high glucose uptake, the combination of PRRT and chemotherapy in parallel constitutes a novel therapeutic scheme. The chemotherapeutic drugs used as PRRT radiosensitizing factors are mainly 5-fluorouracil (5- FU), capecitabine (CAP), and temozolomide (TEM). Several trials have recently demonstrated prime results of this combination in terms of treatment efficacy and generally mild toxicities [47,54,57,58,59,60]. However, there is still no formal guideline that supports its use in specific contexts of NET disease characteristics due to limited data and the small size of the series of patients so far. The efficacy and safety assessment of this novel strategy, as well as the role of ^18^F-FDG PET in this setting, was not within the scope of the present systematic review and meta-analysis.

The complementary role of ^68^Ga-DOTA-TOC/TATE/NOC-PET and ^18^F-FDG PET/CT has previously been investigated across different NET primaries treated with regimens other than just PRRT [61,62]. Dual functional imaging provides relevant information regarding tumor behavior and aggressiveness, therefore, favoring a more personalized treatment strategy, resolving limitations linked to both histopathologic grading, and tumor heterogeneity [62,63]. Importantly, ^18^F-FDG PET/CT has demonstrated a high positive predictive value in identifying G2 NETs [64]. While assessing the prognosis and tumor aggressiveness by means of dual-functional imaging, treatment strategies could be adapted accordingly, as semiquantitative parameters, such as SUV at ^18^F-FDG PET/CT seem to be reliable markers to guide treatment decisions in this context [65]. Therefore, apart from predicting PRRT response, ^18^F-FDG parameters could be considered for NET characterization, especially in the subset of NETs with less affinity for ^68^Ga-DOTA-TOC/TATE/NOC and higher ^18^F-FDG uptake to optimize disease prognostication and prediction of tumor aggressiveness, as these features are currently substantiated mainly from histopathologic evaluation [63,65].

Our study has some limitations. Evolving functional imaging modalities targeting SSTRs along with recent classification changes in NEN histopathology and the utilization of ^18^F-FDG PET mainly in higher grade NETs, may all have affected the selection of eligible patients for ^18^F-FDG PET imaging prior to PRRT administration. Moreover, our study constitutes a subset analysis of multiple cohort studies on NET patients treated with PRRT, not always designed to assess differences in ^18^F-FDG PET status. In addition, the included studies were mostly retrospective, and selection bias is very likely due to the assignment of patients for ^18^FDG-PET prior to PRRT based on age, performance status, primary tumor site, grade, extent of metastatic disease, and prior treatments. Furthermore, the variation in the number of administered PRRT cycles in the included studies may have affected our findings, as there is evidence of a proportional correlation between the dose received by the tumor and the PRRT response outcome [32,33,38,66]. Other limitations were the ambiguity over FDG SUVmax cut-offs and considerable tumor heterogeneity observed across the included studies. In the absence of high-quality randomized controlled studies, we performed a systematic review and meta-analysis summarizing currently available evidence by applying a comprehensive search strategy, as well as a validated quality assessment protocol of the included cohort studies. We could highlight the importance of ^18^F-FDG PET as a means to detect metabolically active lesions and predict PRRT response and survival outcomes in NET patients. Upregulated glucose metabolism may indeed reflect a different radiosensitivity to PRRT possibly related to the activation of proliferating pathways that could render the tumor less prone to respond to PRRT or even more likely to relapse shortly after PPRT administration.

## 5. Conclusions

The present study provides a systematic review and meta-analysis focusing on the clinical utility of ^18^F-FDG PET imaging in patients with NETs, as a predictive tool for PRRT administration. Although relatively favorable outcomes are evident in ^18^F-FDG PET-positive patients with disease control rates as high as 74%, we could confirm a higher risk for progression and death in this subset. Therefore, there might be a place for ^18^F-FDG PET imaging as a predictive tool for PRRT administration in the management of patients with NETs. Dual functional imaging should probably be considered prior to PRRT initiation to delineate tumor SSTR expression and glycolytic metabolic activity in the context of a personalized treatment strategy. Thus, baseline dual-functional imaging assessment of NETs could be used for the selection of patients requiring PRRT or other systemic treatments as well as the prognostic evaluation of the disease with potential implications on the intensity of the surveillance strategy. Our results should be interpreted with caution though, due to the potential selection bias of the included studies. Further well-designed randomized controlled trials with the aim to assess the clinical utility of ^18^F-FDG PET imaging across a wide range of NET primaries and lower-grade tumors are warranted. Such studies should aim to evaluate the role of dual-functional imaging in disease prognosis, but also as a predictive tool in respect of available NET treatments in order to identify patients who will most benefit from the utilization of ^18^F-FDG PET imaging.

## Figures and Tables

**Table 1:** Characteristics of the included studies.

Adult Studies	Study Design	Primary NET Site	WHO Grade	No of Patients, FDG (−): FDG(+)	DCR (%) FDG (−): FDG(+)	mPFS (months) FDG (−): FDG(+)	mOS (months) FDG (−): FDG(+)	SUVmax Cut-Off
Adnan et al. [12].	Single-Centre Retrospective Cohort Study	13T, 14M	All Grades	1:26	48.1 *	36 *	66 *	N/S
Adnan et al. [32].	Single-Centre Retrospective Cohort Study	39GEP, 6L, 1T, 1Other, 12UPO	G1 and G2	13:46	92.3:67.4	70.7:26.8	79.3:5.5	N/S
Nilica et al. [36].	Single-Centre Retrospective Cohort Study	24SI, 20P,2R, 2C, 1S, 8L, 9UPO	All Grades	37:29	81: 41.4	N/A	N/A	3
Sansovini et al. [33].	Single-Centre Prospective Phase II Study	55P	G1 and G2	23:32	95.6:78.1	68.7:21.2	NR:63.8	2.5
Severi et al. [34].	Single-Centre Retrospective Cohort Study	12SI, 29P, 2R, 2C, 1L, 10UPO	G1 and G2	19:33	100:75.7	32:20	N/A	2.5
Thapa et al. [37].	Single-Centre Retrospective Cohort Study	12SI, 21P, 9R, 1S, 7UPO	All Grades	33:17	96.9:52.9	N/A	N/A	N/S
Zemczak et al. [35].	Multi-Centre Retrospective Study	22SI, 24P, 16C,4L, 9UPO	G1 and G2	48:27	85.1:83.3	59.3:22.2	NR:55.8	N/S
Zhang et al. [38].	Single-Centre Retrospective Cohort Study	139SI, 199P, 20R, 8S, 38L, 42Other, 49UPO	All Grades	113:382	N/A	24.1:18.5	83.1:53.2	N/S
Chan et al. [40].	Single-Centre Retrospective Cohort Study	24SI, 14P, 11Other	All Grades	49 *	N/A	26.6:19.7	N/A	4
Kunikowska et al. [42].	Single-Centre Prospective Cohort Study	N/S	G1 and G2	11:7	81.8:0	NR:11.7	NR in both groups	N/S
Sitani et al. [39].	Single-Centre Retrospective Cohort Study	112SI, 42C, 142P, 16S, 58L/T/M, 10Other, 88 UPO	All Grades	299:169	93.3:84.6	N/A	N/A	5
Binderup et al. [41].	Single-Centre Retrospective Cohort Study	90SI, 37P, 12C, 27UPO	All Grades	39:39	N/A	31.2 *	75.6 *	N/S

Abbreviations: C: Colon; DCR: Disease Control Rate; FDG: fluorodeoxyglucose; G1: Grade 1; G2: Grade 2; GEP: Gastroenteropancreatic; L: Lung; M: Mediastinum; mOS: median Overall Survival; mPFS: median Progression-Free Survival; NET: Neuroendocrine Tumor; N/A: Not Available; NR: Not Reached; N/S: not specified; P: Pancreas; PRRT: Peptide Receptor Radionuclide Therapy; R: Rectum; S: Stomach; SI: Small Intestine; SUVmax; Maximum Standardized Uptake Value; T: Thymus; UPO: Unknown Primary Origin; WHO: World Health Organization. * In the whole study cohort. Details on FDG strata are not provided. FDG(−) and FDG(+) stands for “not FDG-avid” and “FDG-avid” tumor lesions, respectively.

## Data Availability

Individual patient data from the original studies included in the present meta-analysis is not available and data sharing at this level is not applicable for a systematic review.

## Supplementary Materials

The following are available online at https://www.mdpi.com/article/10.3390/cancers13081813/s1, Figure S1A: Egger’s plot for studies included in the comparison of FDG(-) vs. FDG(+) NET patients receiving peptide receptor radionuclide therapy (PPTR) with respect to disease control rate (DCR)., and 1B: Galbraith’s plot for studies included in this analysis; Figure S2A: Egger’s plot for studies included in the comparison of FDG(−) vs. FDG(+) NET patients receiving peptide receptor radionuclide therapy (PPTR) with respect to adjusted hazard ratios (HRs) progression-free survival analysis, and 2B: Galbraith’s plot for studies included in this analysis; Figure S3: Egger’s plot for studies included in the comparison of FDG(−) vs. FDG(+) NET patients receiving peptide receptor radionuclide therapy (PPTR) with respect to adjusted hazard ratios (HRs) overall survival analysis. Table S1: Systematic literature search strategy; Table S2. Newcastle-Ottawa Scale (NOS) template.
