# Supplementary material for: Clinical Utility of 18F-FDG PET in Neuroendocrine Tumors Prior to Peptide Receptor Radionuclide Therapy: A Systematic Review and Meta-Analysis
Source: Cancers (Basel). 2021 Apr 10;13(8):1813. doi: 10.3390/cancers13081813 (PMC8069875; doi:10.3390/cancers13081813)
Supplement: Supplementary file 1 [file cancers-13-01813-s001.pdf]

cancers MDPI

Table S1: Systematic literature search strategy

Medline via Ovid 200624

	Search rerms	Number
	**Neuroendocrine tumors**	
**1.**	(neuroendocrine adj4 (tumo?r* or neoplas* or cancer* or carcinom* or malignanc*)).ab,kf,ti.	**22,028**
**2.**	neuroendocrine tumors/ or adenoma, acidophil/ or adenoma, basophil/ or adenoma, chromophobe/ or apudoma/ or carcinoid tumor/ or malignant carcinoid syndrome/ or carcinoma, neuroendocrine/ or somatostatinoma/ or vipoma/ or Multiple Endocrine Neoplasia Type 1/ or carcinoma, medullary/ or carcinoma, merkel cell/ or exp neurilemmoma/ or exp paraganglioma/ or pheochromocytoma/	**76,309**
**3.**	((carcinoma* adj2 medulla*) or (cancer adj2 medulla*) or (tumo?r* adj2 medulla*)).ab,kf,ti.	**8,903**
**4.**	(carcinoid* or somatostatinoma* or vipoma* or apudoma* or (adenoma* adj2 chromophobe) or (adenoma* adj2 basophil*) or (adenoma* adj2 acidophil*) or "Multiple Endocrine Neoplasia Type 1" or "MEN 1" or Neurilemmoma* or Neurilemoma* or Schwannoma* or Neurinoma* or Schwannomatosis or Schwannomatoses or Paraganglioma* or Pheochromocytoma*).ab,kf,ti.	**61,149**
**5.**	(merk* adj4 (tumo?r* or cancer or carcinom*)).ab,kf,ti.	**3,411**
**6.**	("Gastro-enteropancreatic neuroendocrine tumor" or "Thyroid cancer, medullary").mp. [mp=title, abstract, original title, name of substance word, subject heading word, floating sub-heading word, keyword heading word, organism supplementary concept word, protocol supplementary concept word, rare disease supplementary concept word, unique identifier, synonyms] **Obs! Fraserna söks i fält MP för att de finns som Supplementary Concept i MeSH**	**1,294**
**7.**	1 or 2 or 3 or 4 or 5 or 6	**107,828**
	**Lutetium**	
**8.**	Lutetium/	**902**
**9.**	(lutetium or edotreotide).mp.	**2,052**
**10.**	(radiopeptide* or dotatoc or DOTATATE or PRRT or "peptide receptor radionuclear therapy" or OCTREOTATE or DOTA or 177Lu or Lu177 or "Lu-177" or "177-Lu").ab,kf,ti.	**6,162**
**11.**	8 or 9 or 10	**7,111**
	**Combined sets**	
**12.**	7 AND 11	**1,841**

Embase via embase.com 200624

	Search terms	Number
	**Neuroendocrine tumors**	
**1.**	(neuroendocrine NEAR/4 tumo$r*):ti,ab,kw	**29,104**
**2.**	(neuroendocrine NEAR/4 cancer*):ti,ab,kw	**3,100**
**3.**	(neuroendocrine NEAR/4 neoplas*):ti,ab,kw	**4,240**
**4.**	(neuroendocrine NEAR/4 carcinom*):ti,ab,kw	**9,793**
**5.**	(neuroendocrine NEAR/4 malignanc*):ti,ab,kw	**621**
**6.**	(adenoma* NEAR/2 acidophil):ti,ab,kw	**33**
**7.**	(adenoma* NEAR/2 basophil):ti,ab,kw	**28**
**8.**	(adenoma* NEAR/2 chromophobe):ti,ab,kw	**590**
**9.**	'gastroenteropancreatic neuroendocrine tumor'/exp OR 'neuroendocrine carcinoma'/exp OR 'paraganglioma'/de OR 'carotid body tumor'/de OR 'thyroid carcinoma'/de OR 'bronchus carcinoid'/de OR 'carcinoid syndrome'/de OR	**128,860**

	'gastrointestinal carcinoid'/exp OR 'apudoma'/de OR 'vipoma'/de OR 'somatostatinoma'/de OR 'medullary carcinoma'/de OR 'thyroid medullary carcinoma'/de OR 'neurilemoma'/de OR 'adrenal medulla tumor'/de OR 'pheochromocytoma'/de OR 'neuroendocrine tumor'/de OR 'multiple endocrine neoplasia type 1'/de	
**10.**	carcinoid*:ti,ab,kw OR somatostatinoma*:ti,ab,kw OR vipoma*:ti,ab,kw OR apudoma*:ti,ab,kw OR neurilemmoma*:ti,ab,kw OR neurilemoma*:ti,ab,kw OR schwannoma*:ti,ab,kw OR neurinoma*:ti,ab,kw OR schwannomatosis:ti,ab,kw OR schwannomatoses:ti,ab,kw OR paraganglioma*:ti,ab,kw OR pheochromocytoma*:ti,ab,kw OR 'multiple endocrine neoplasia type 1':ti,ab,kw OR 'men 1':ti,ab,kw	**79,754**
**11.**	((carcinoma* NEAR/2 medulla*):ti,ab,kw) OR ((cancer NEAR/2 medulla*):ti,ab,kw) OR ((tumo$r* NEAR/2 medulla*):ti,ab,kw)	**11,844**
**12.**	(merk* NEAR/4 tumo$r*):ti,ab,kw	**662**
**13.**	(merk* NEAR/4 cancer):ti,ab,kw	**241**
**14.**	(merk* NEAR/4 carcinom*):ti,ab,kw	**4,621**
**15.**	1 OR 2 OR 3 OR 4 OR 5 OR 6 OR 7 OR 8 OR 9 OR 10 OR 11 OR 12 OR 13 OR 14	**175,784**
	**Lutetium**	
**16.**	'lutetium'/de OR 'lutetium 177'/de OR 'edotreotide'/de	**5,303**
**17.**	lutetium:ti,ab,kw OR lutetium177:ti,ab,kw OR edotreotide:ti,ab,kw OR radiopeptide*:ti,ab,kw OR dotatoc:ti,ab,kw OR dotatate:ti,ab,kw OR prrt:ti,ab,kw OR 'peptide receptor radionuclear therapy':ti,ab,kw OR octreotate:ti,ab,kw OR dota:ti,ab,kw OR 177lu:ti,ab,kw OR lu177:ti,ab,kw OR 'lu-177':ti,ab,kw OR '177-lu':ti,ab,kw	**11,630**
**18.**	16 OR 17	**13,488**
	**Combined sets**	
**19.**	15 AND 18	**4,273**
	**Excluded: confererence abstracts, editorials, letters, notes**	
**20.**	15 AND 18	**2,162**

Cochrane via Wiley 200624

	Search terms	Number
	**Neuroendocrine tumors**	
**1.**	(neuroendocrine NEAR/4 (tumo*r* OR neoplas* OR cancer* OR carcinom* OR malignanc*))	**910**
**2.**	MeSH descriptor: [Neuroendocrine Tumors] this term only	**155**
**3.**	MeSH descriptor: [Adenoma, Acidophil] this term only	**0**
**4.**	MeSH descriptor: [Adenoma, Basophil] this term only	**0**
**5.**	MeSH descriptor: [Adenoma, Chromophobe] this term only	**1**
**6.**	MeSH descriptor: [Apudoma] this term only	**0**
**7.**	MeSH descriptor: [Carcinoid Tumor] this term only	**85**
**8.**	MeSH descriptor: [Malignant Carcinoid Syndrome] this term only	**40**
**9.**	MeSH descriptor: [Carcinoma, Neuroendocrine] this term only	**47**
**10.**	MeSH descriptor: [Somatostatinoma] this term only	**3**
**11.**	MeSH descriptor: [Vipoma] this term only	**2**
**12.**	MeSH descriptor: [Multiple Endocrine Neoplasia Type 1] this term only	**8**
**13.**	MeSH descriptor: [Carcinoma, Medullary] this term only	**13**
**14.**	MeSH descriptor: [Carcinoma, Merkel Cell] this term only	**17**
**15.**	MeSH descriptor: [Neurilemmoma] explode all trees	**72**
**16.**	MeSH descriptor: [Paraganglioma] explode all trees	**63**

**17.**	MeSH descriptor: [Pheochromocytoma] this term only	**60**
**18.**	2 OR 3 OR 4 OR 5 OR 6 OR 7 OR 8 OR 9 OR 10 OR 11 OR 12 OR 13 OR 14 OR 15 OR 16 OR 17	**428**
**19.**	(((carcinoma* NEAR/2 medulla*) OR (cancer NEAR/2 medulla*) OR (tumo*r* NEAR/2 medulla*))):ti,ab,kw	**167**
**20.**	((carcinoid* OR somatostatinoma* OR vipoma* OR apudoma* OR (adenoma* NEAR/2 chromophobe) OR (adenoma* NEAR/2 basophil*) OR (adenoma* NEAR/2 acidophil*) OR "multiple endocrine neoplasia type 1" OR "MEN 1" OR neurilemmoma* OR neurilemoma* OR schwannoma* OR neurinoma* OR schwannomatosis OR schwannomatoses OR paraganglioma* OR pheochromocytoma*)):ti,ab,kw	**931**
**21.**	((merk* NEAR/4 (tumo*r* OR cancer OR carcinom*))):ti,ab,kw	**74**
**22.**	1 OR 18 OR 19 OR 20 OR 21	**1,877**
	**Lutetium**	
**23.**	MeSH descriptor: [Lutetium] this term only	**1**
**24.**	(lutetium OR edotreotide):ti,ab,kw	**82**
**25.**	(radiopeptide* OR dotatoc OR dotatate OR prrt OR "peptide receptor radionuclear therapy" OR octreotate OR dota OR 177lu OR lu177 OR "lu-177" OR "177-lu"):ti,ab,kw	**252**
**26.**	23 OR 24 OR 25	**266**
	**Combined sets**	
**27.**	22 AND 26	**132**
	**Excluded: confererence abstracts, editorials, letters, notes**	
**28.**	22 AND 26	**106**

Web of Science via Clarivate 200624

	**Search terms**	**Number**
	**Neuroendocrine tumors**	
**1.**	TS=(neuroendocrine NEAR/3 (tumo$r* or neoplas* or cancer* or carcinom* or malignanc*))	**30,388**
**2.**	TS=((carcinoma* NEAR/1 medulla*) or (cancer NEAR/1 medulla*) or (tumo$r* NEAR/1 medulla*))	**9,259**
**3.**	TS=(carcinoid* or somatostatinoma* or vipoma* or apudoma* or (adenoma* NEAR/1 chromophobe) or (adenoma* NEAR/1 basophil*) or (adenoma* NEAR/1 acidophil*) or "Multiple Endocrine Neoplasia Type 1" or "MEN 1" or Neurilemmoma* or Neurilemoma* or Schwannoma* or Neurinoma* or Schwannomatosis or Schwannomatoses or Paraganglioma* or Pheochromocytoma*)	**58,943**
**4.**	TS=(merk* NEAR/3 (tumo$r* or cancer or carcinom*))	**4,281**
**5.**	1 OR 2 OR 3 OR 4	**92,231**
	**Lutetium**	
**6.**	TS=(lutetium or edotreotide or radiopeptide* or dotatoc or DOTATATE or PRRT or "peptide receptor radionuclear therapy" or OCTREOTATE or DOTA or 177Lu or Lu177 or "Lu-177" or "177-Lu")	**14,244**
	**Combined sets**	
**7.**	5 AND 6	**2,817**
	**Excluded: confererence abstracts, editorials, letters, notes**	
		**2,100**

Table S2. Newcastle-Ottawa Scale (NOS) template.

Adult Studies	Selection	Comparability	Outcome
Adnan et al [22].	***	**	***
Adnan et al [17].	***	*	***
Nilica et al [23].	***	*	***
Sansovini et al [18].	****	**	***
Severi et al [19].	***	*	***
Thapa et al [24].	***	*	***
Zemczak et al [20].	***	*	***
Zhang et al [25].	***	**	***
Chan et al [27].	**	**	***
Kunikowska et al [29].	***	*	***
Sitani et al [26].	***	**	***
Binderup et al [28].	***	**	***

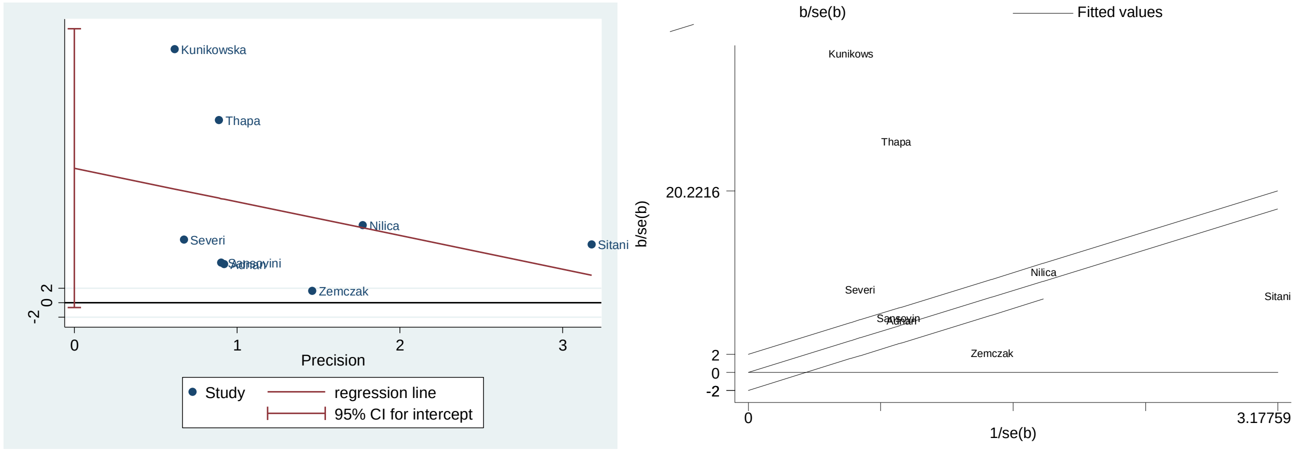

Figure S1A Egger's plot for studies included in the comparison of FDG(-) vs. FDG(+) NET patients receiving peptide receptor radionuclide therapy (PPTR) with respect to disease control rate (DCR)., and S1B: Galbraith's plot for studies included in this analysis

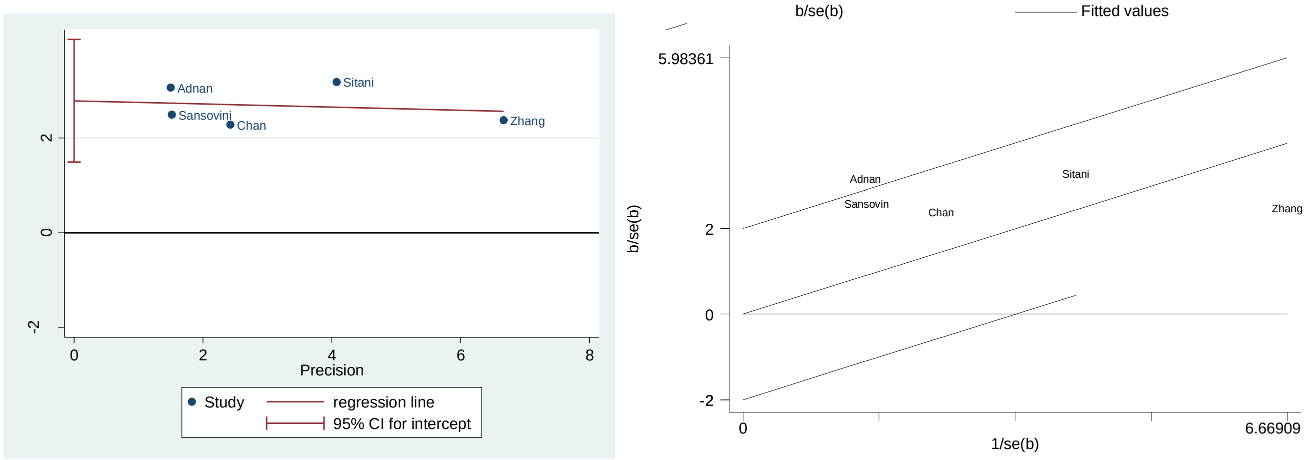

Figure S2A Egger's plot for studies included in the comparison of FDG(-) vs. FDG(+) NET patients receiving peptide receptor radionuclide therapy (PPTR) with respect to adjusted hazard ratios (HRs) progression-free survival analysis, and S2B: Galbraith's plot for studies included in this analysis

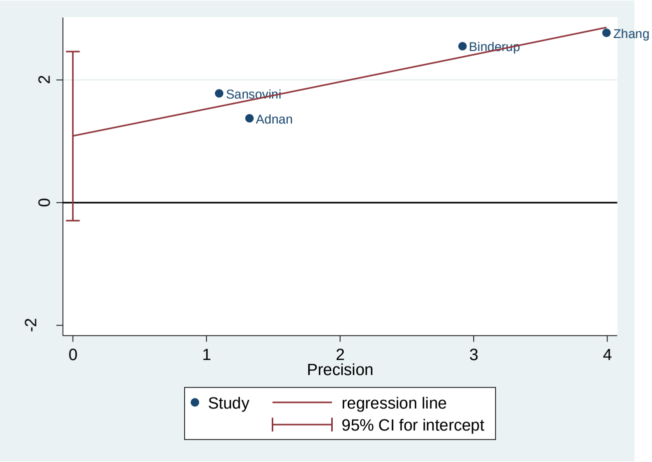

Figure S3 Egger's plot for studies included in the comparison of FDG(-) vs. FDG(+) NET patients receiving peptide receptor radionuclide therapy (PPTR) with respect to adjusted hazard ratios (HRs) overall survival analysis. Table S1: Systematic literature search strategy; Table S2. Newcastle-Ottawa Scale (NOS) template
